# Human Babesiosis Caused by *Babesia duncani* Has Widespread Distribution across Canada

**DOI:** 10.3390/healthcare6020049

**Published:** 2018-05-17

**Authors:** John D. Scott, Catherine M. Scott

**Affiliations:** International Lyme and Associated Diseases Society, Bethesda, MD 20827, USA; kittjohn365@gmail.com

**Keywords:** human babesiosis, *Babesia duncani*, babesial piroplasm, incidence, epidemiology, ticks, vectors, Canada

## Abstract

Human babesiosis caused by *Babesia duncani* is an emerging infectious disease in Canada. This malaria-like illness is brought about by a protozoan parasite infecting red blood cells. Currently, controversy surrounds which tick species are vectors of *B. duncani.* Since the availability of a serological or molecular test in Canada for *B. duncani* has been limited, we conducted a seven-year surveillance study (2011–2017) to ascertain the occurrence and geographic distribution of *B. duncani* infection country-wide. Surveillance case data for human *B. duncani* infections were collected by contacting physicians and naturopathic physicians in the United States and Canada who specialize in tick-borne diseases. During the seven-year period, 1119 cases were identified. The presence of *B. duncani* infections was widespread across Canada, with the highest occurrence in the Pacific coast region. Patients with human babesiosis may be asymptomatic, but as this parasitemia progresses, symptoms range from mild to fatal. Donors of blood, plasma, living tissues, and organs may unknowingly be infected with this piroplasm and are contributing to the spread of this zoonosis. Our data show that greater awareness of human babesiosis is needed in Canada, and the imminent threat to the security of the Canadian blood supply warrants further investigation. Based on our epidemiological findings, human babesiosis should be a nationally notifiable disease in Canada. Whenever a patient has a tick bite, health practitioners must watch for *B. duncani* infections, and include human babesiosis in their differential diagnosis.

## 1. Introduction

Human babesiosis, a zoonosis, is an infectious disease caused by intraerythrocytic protozoan parasites of the genus *Babesia* [1]. These microscopic malaria-like organisms (1−3 µm) invade human erythrocytes and lyse them, causing a febrile hemolytic anemia [2]. In the continental United States and Canada, *Babesia duncani* and *Babesia microti* (Apicomplexa: Babesiidae) are commonly recognized and identified in humans. These apicomplexan piroplasms are morphologically similar but are genetically different [3]. Both babesial strains reside in red blood cells [2]. Pathologically, *Babesia* in humans ranges from asymptomatic or self-limiting, to mild febrile illness, to fatal, especially in the elderly, immunocompromised, and splenectomized patients [1]. Clinical symptoms associated with human babesiosis include sweats (night or day), chills, profound fatigue, malaise, fever, increased thirst, frequent urination, headaches, body aches, sleep disturbance, cognitive impairment, and depression [1,4]. *Babesia* has four life stages, namely sporozoite, trophozoite, merozoite, and gamete. Trophozoites and merozoites occur in mammalian cells, whereas sporozoites are in tick salivary glands and gametes are in the tick midgut [5]. Since sporozoites reside in the tick salivary glands, they can be transmitted promptly to its host when the tick begins to feed [5].

It is noteworthy that *Babesia* hemoparasites have adapted well to the Nearctic zoogeographic region. In the Neotropics, *Babesia* species have circulated in enzootic transmission cycles involving ixodid (hard-bodied) ticks (Acari: Ixodidae) and vertebrate hosts dating back 20−30 million years [6]. In 1957, the first case of human piroplasmosis, a zoonotic disease, was reported in a cattle farmer who came from the village of Strmec in Croatia [7]. This geographic area has *Ixodes ricinus* ticks, which are known to transmit *Babesia* piroplasms [8]. 

In North America, *B. duncani* is detected and diagnosed continent-wide. Although there is substantive evidence that certain ixodid ticks (i.e., *Ixodes angustus*, *Ixodes muris*, *Ixodes pacificus*, *Ixodes scapularis*, and *Ixodes spinipalpis*) are vectors of *B. duncani*, this fact has not been definitively established [9]. Depending on the species of *Babesia*, this zoonotic parasite can be transmitted in particular ticks by transovarial transmission (female to eggs to larvae) and, also, via transstadial transmission (larvae to nymphs to adults) [5].

Hersh et al. detected *B. microti* in blacklegged ticks, *I. scapularis*, collected from small mammals, meso-mammals, sciurids, and songbirds [10]. Migratory songbirds, in particular, widely disperse ticks infected with babesial piroplasms and, during northward spring migration, transport bird-feeding ticks into Canada from as far south as Brazil [11,12]. These findings indicate that certain avian hosts are reservoirs; however, songbird-transported nymphs have previously had a blood meal from a previous host, so the source of infection is not clear. Not only is *I. scapularis* a vector of at least 10 zoonotic, tick-borne pathogens, it is also a competent vector of many of these pathogens, including *B. microti*. Based on the fact that *B. duncani* is morphologically similar to *B. microti*, there is reasonable likelihood that *I. scapularis* is a vector of *B. duncani*. For example, a fully engorged *I. scapularis* nymph was collected from a human patient who was subsequently found to have a *B. duncani* infection [13], and this parasitism suggests that *I. scapularis* is a vector of *B. duncani*. 

Until recently, the presence of *B. duncani* has been a mystery in Canada. This *Babesia* species has been lurking in woodlands and arboreal areas country-wide and, for the most part, has gone unnoticed. The National Microbiology Laboratory, Public Health Agency of Canada, Health Canada does not have a serological or molecular test to specifically detect *B. duncani*. Additionally, Health Canada does not have an approved blood donor screening assay for *Babesia* piroplasms. In the United States of America, human babesiosis is a nationally notifiable disease; however, in Canada, it is not. Notably, serological and nucleic acid tests have recently been approved in the USA to detect *B. microti* in samples of whole blood, blood components, living organs, and tissue specimens from volunteer donors [14]. 

Both *B. duncani* and *B. microti* have been detected in Canadian patients who have no history of out-of-province travel. Scott [13] documented a case of *B. duncani* in a husband and wife who had no history of out-of-province travel and had not had blood transfusions. In addition, Bullard et al. [15] reported a *B. microti* infection in a boy residing in Manitoba, who had no history of out-of-province travel, and had not had a blood transfusion. Some medical personnel contend that people must visit an endemic area to contract human babesiosis while other epidemiologists reveal that this longstanding stance is presumptive. The aim of this pilot study was to determine the occurrence and geographic distribution of *B. duncani* infections acquired locally across Canada.

## 2. Materials and Methods

In order to get a representative sample of *B. duncani* cases, we contacted 20 medical practitioners, namely physicians (*n* = 10) and naturopathic physicians (*n* = 10) across southern Canada and the northern USA whose focus in their medical practice is tick-associated diseases. These healthcare providers were specifically asked to provide the number of Canadian patients with *B. duncani* infections. These data were then used to tabulate the occurrence of *B. duncani* infections in each province of Canada.

Since *B. duncani* infections are not reportable in Canada, we obtained the majority of the preliminary data from medical professionals in the USA. In order for patients to meet the inclusion criteria, they had to have serological and/or molecular positivity for *B. duncani*. All laboratories had to meet the quality control standards of the Clinical Laboratory Improvement Amendments (CLIA), which ensure quality laboratory testing performance. The laboratories used for *Babesia* testing were IGeneX Laboratory, LabCorp, Medical Diagnostic Laboratory, Quest Diagnostics, and County of Sonoma Public Health Laboratory. These laboratories are internationally and/or nationally accredited. In addition, these laboratories have passed the strict testing guidelines of several states, and are certified by the U.S. government via the Centers for Medicare and Medicaid Services. Each laboratory had serological and molecular testing for the WA1 strain (*B. duncani*). For serological testing of *B. duncani*, the *B. duncani* WA1 IgG assay was performed by the immunofluorescence antibody (IFA) method [16]. For molecular testing of *B. duncani*, the RNA probe using the internal transcribed spacer (ITS) regions of the nuclear ribosomal RNAs method was employed [17].

The U.S. doctors were specifically asked for the number of Canadian patients who were diagnosed with human babesiosis, caused by *B. duncani*, and their provincial residency in Canada. Patients were also required to have typical clinical symptoms associated with human babesiosis. Although not required, supportive information for clinical assessment included whether a patient had a blood transfusion and/or outdoor exposure to grassy and wooded areas.

Ethical approval was not required because no personal identifiable information was being collected. Healthcare professionals provided only anonymous, aggregate information on the numbers of patients who met the study inclusion criteria.

## 3. Results

**Figure 1:**
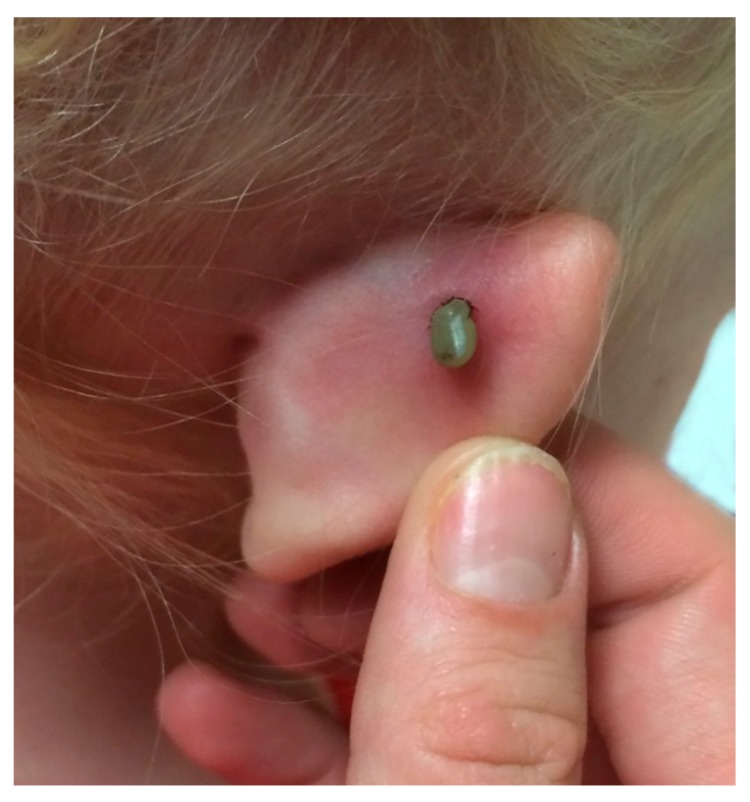
A blacklegged tick female parasitizes the ear of a seven-year-old girl. Based on the size of this partially engorged female, it had been feeding for four days. When the tick bites its host, the hypostome secretes a compound that anesthetizes the skin at the point of entry. Consequently, the majority of patients do not recall a tick bite.

Overall, 1119 cases of *B. duncani* were reported by physicians and naturopathic physicians during the seven-year period (2011–2017) (Table 1). The geographic distribution of *B. duncani* cases extended from coast to coast (Table 1). Clinically, 89% of these Canadian patients were diagnosed in United States, while 11% were diagnosed in Canada. Based on the fact that other *Ixodes* ticks in other zoogeographic regions in the northern hemisphere transmit *Babesia* species to humans, we suggest that *B. duncani* can be transmitted to humans in North America via a tick bite (Figure 1). Epidemiologically, *B. duncani* infections are apparent in various regions across Canada, so physicians and naturopathic physicians should consider them in their differential diagnosis.

## 4. Discussion

*Babesia duncani* is an emerging tick-borne pathogen in Canada. Our pilot study represents the first documentation in Canada of *B. duncani* infections coast to coast. We know of no previous study reporting this infectious disease information in Canada. With respect to any limitations on our study, we were not privy to individual patients’ files. We relied on the integrity of the medical professionals to provide the number of cases that met the inclusion criteria. As a result, these clinical cases provide compelling evidence that *B. duncani* infections are nationwide.

In this collaborative study, we recognize that there are limitations in surveying the entire Canadian population. Since we only enlisted 20 physicians and naturopathic physicians, we would have missed some *B. duncani* cases. One important limitation was the fact that there was no testing for *B. duncani* available in Canada. Another clinical limitation was that some *B. duncani* cases will be in the asymptomatic phase, and thus not recognized by medical professionals. Although there can be inexactitudes in the diagnoses for *B. duncani* infection, we are reassured that the physicians and naturopathic physicians who participated in this study had specialized training in tick-borne diseases, and provided proficient and forthright diagnoses. Not only did medical practitioners assess patients for signs and symptoms, they also employed serological and molecular testing to support their diagnoses. Since *B. duncani* infections can be subclinical in the early stage, especially in young people, we must be open-minded as to the origin of infection. Because migratory songbirds transport *Babesia*-infected ticks hundreds of kilometers, determining the actual source of infection is difficult. Despite our conservative findings, we provide a substantive representation of *B. duncani* cases across Canada.

During this study, physicians and naturopathic physicians reiterated that *B. duncani* is harder to treat than *B. microti*, and typically requires longer antibabesiosis treatment. In some cases, *Babesia* infections can be refractory, and recrudescence of infection may occur [18]. It appears that one of the pleomorphic forms of *B. duncani* promptly goes into the dormant phase upon the initiation of anti-piroplasmic treatment. When the antimicrobial regimen ends, the dormant stage reverts to the active form. Re-treatment may be warranted to ambush this recalcitrant form. 

In either the tick or the host, *B. duncani* may be a single pathogen or it may co-mingle with the Lyme disease bacterium, *Borrelia burgdorferi*, or another tick-associated pathogen. When patients have Lyme disease and are co-infected, the most common tick-borne pathogen is *Babesia* [19,20,21,22,23,24,25,26]. The geographic distribution of babesiosis and Lyme disease is often sympatric, and the causal organisms of both diseases share the same vector and reservoir hosts. Up to 56% of patients with babesiosis in the northeastern USA, in particular, the easternmost part of Long Island, had evidence of concurrent Lyme disease [19,24,27,28]. Therefore, whenever a person is bitten by an *Ixodes* species tick, healthcare practitioners should carefully assess patients’ symptoms and screen for several zoonotic, tick-borne pathogens. When a tick bites its host, it first anesthetizes the skin at the point of entry (punctum), and patients often do not remember being bitten. In fact, one tick-host-pathogen study reported that only 14% of patients recall a tick bite [29].

An attached tick can be overlooked for several days or be completely missed (Figure 1). For example, a four-year-old girl from Pennsylvania was hospitalized with lower extremity weakness and unsteady gait; the attending physician overlooked the attached tick [30]. She was in hospital for eight days with flaccid paralysis, facial weakness, and complete areflexia before magnetic resonance imaging on her head revealed an attached, fully engorged female of the American dog tick, *Dermacentor variabilis*. Of note, fully engorged females of certain tick species (i.e., *D. variabilis*, *I. pacificus*, and *I. scapularis*) will cause tick paralysis. 

Controversy abounds about which tick species are competent vectors of *B. duncani*. One team of U.S. researchers purported that the lone star tick, *Amblyomma americanum*, is a vector of *Babesia* [31]. In addition, other researchers have suggested that the *I. pacificus* and *I. spinipalpis* are vectors in far-western North America and, in central and eastern Canada, *I. scapularis* are vectors of *B. duncani*. All of these tick species feed on rodents, are transported into Canada by Neotropical and southern temperate songbirds during northward spring migration, and act as a potential source of *B. duncani* [32,33]. Ultimately, vector competency studies are needed to determine whether these tick species are competent vectors of *B. duncani.*

*Babesia duncani* is present continent-wide, and its occurrence will vary between regions and within regions. Prince et al. conducted a two-year study of blood donor specimens collected from diverse geographic areas across U.S.A., and found that *B. duncani* is approximately five times more apparent than *B. microti* [16]. These findings counter the long-standing perception that *B. duncani* is confined to the West Coast. With only a national border between the U.S.A. and Canada, our findings are consistent with their babesial results. Additionally, one team of clinicians reported *B. duncani* along the entire eastern U.S.A. from Florida to Maine [25]. Since there have been no previous pathologic studies of *B. duncani* in Canada, the rate of subclinical infection compared to apparent infection is unknown. Moreover, we do not know if patients with low income have had to forfeit testing for *B. duncani* because of personal cost. 

Transfusion-transmitted babesiosis has been quickly increasing in occurrence across North America since the start of the 21st century [34,35]. A study of transfusion-transmitted babesiosis in blood donors across central and eastern Canada found a low prevalence of *B. microti* [36]; however, there was no screening for *B. duncani*. Moreover, these researchers tested the ticks for *B. microti*, but did not test them for *B. duncani.* An increasing number of human babesiosis cases has been caused by blood transfusions from donors with a subclinical infection [37]. Also, pertinent to our babesial study, *B. duncani* has been transmitted by blood transfusion [38,39]. Since blood donors are often unaware that they are infected, the number of transfusion-transmitted reported cases has increased [39]. Blood products collected in *Babesia*-endemic areas are disseminated widely, and clinicians in nonendemic areas may fail to include human babesiosis in the differential diagnosis of patients who have had a recent blood transfusion. Notably, blood transfusions are the most common mode of acquisition of neonatal babesiosis [1,37,40]. Canadian Blood Services has not been routinely screening for *Babesia* piroplasms; however, based on the occurrence of *B. duncani*, blood donors should be screened in order to prevent the occurrence of *B. duncani* transmission to human recipients. Blood donors in the USA have not been screened routinely for human babesiosis; however, there are now Food and Drug Administration (FDA)-approved serological and molecular tests to detect *B. microti* in the blood supply [14]. In all likelihood, *B. duncani* will most likely be included. Because donors of blood, plasma, living organs, and tissues can be asymptomatic and have very low parasitemia, they need to be screened for *B. duncani* and *B. microti* [14].

Clinicians have reported perinatal babesiosis and confirmed that *Babesia* piroplasms can be transplacentally transmitted [41,42]. Synchronously, congenital human babesiosis takes place between infected mothers and their neonates [43].

Worldwide, there are at least 100 *Babesia* spp., and they all have a genetically different profile. Although cross-reactivity could be an issue with babesial serology, it would be highly unlikely with a molecular test. The sensitive and species-specific digital droplet PCR assays, which detect and differentiate between *B. duncani* and *B. microti* within ITS regions of the nuclear ribosomal RNAs, provide confirmatory evidence for these two *Babesia* species. Since there have been no species-specific, serological and molecular tests for *B. duncani* in Canada, patients have had to rely on tests validated in the USA, or abroad.

## 5. Conclusions

In this seven-year pilot study, a total of 1119 cases of *B. duncani* infection were detected; the highest number of cases was reported within the Pacific coastal region, where this piroplasm was originally discovered. Based on the recalcitrant nature of *B. duncani*, the authors recommend that Canadian patients with clinical symptoms of human babesiosis be encouraged to have a complete *Babesia* panel to differentiate between *B. duncani* and *B. microti*. With the widespread occurrence of human babesiosis coast to coast, this piroplasmosis warrants being made a nationally notifiable disease in Canada. The optimum antibabesiosis treatment for persistent *B. duncani* infection remains to be determined. Our findings reveal that *B. duncani* is more apparent across Canada than previously elucidated by early American studies, and is not limited to the Pacific Northwest. New public health assessment strategies are needed to counteract the increased number of *B. duncani* infections across Canada.

## Figures and Tables

**Table 1:** The occurrence of *Babesia duncani* infections across Canada, 2011–2017.

By Province	Cases	By Year	Cases
Alberta	25	2011	119
British Columbia	377	2012	145
Manitoba	41	2013	146
New Brunswick	10	2014	147
Nova Scotia	8	2015	162
Newfoundland and Labrador	4	2016	182
Ontario	365	2017	198
Prince Edward Island	7		
Québec	269		
Saskatchewan	13		
Totals	1119		1119
